# A Predictive Energy Management Strategy for Multi-Energy Source Vehicles Based on Full-Factor Trip Information

**DOI:** 10.3390/s22010023

**Published:** 2021-12-22

**Authors:** Fenglai Yue, Qiao Liu, Yan Kong, Junhong Zhang, Nan Xu

**Affiliations:** 1 State Key Laboratory of Engines, Tianjin University, 135 Yaguan Rd., Tianjin 300350, China; yuefenglai@tju.edu.cn; 2 State Key Laboratory of Automotive Simulation and Control, Jilin University, Changchun 130025, China; liuqiao20@mails.jlu.edu.cn (Q.L.); kongyan19@mails.jlu.edu.cn (Y.K.); nanxu@jlu.edu.cn (N.X.)

**Keywords:** global optimization, real-time application, full-factor trip information, multi-energy source vehicles

## Abstract

To achieve the real-time application of a dynamic programming (DP) control strategy, we propose a predictive energy management strategy (PEMS) based on full-factor trip information, including vehicle speed, slip ratio and slope. Firstly, the prediction model of the full-factor trip information is proposed, which provides an information basis for global optimization energy management. To improve the prediction’s accuracy, the vehicle speed is predicted based on the state transition probability matrix generated in the same driving scene. The characteristic parameters are extracted by a feature selection method taken as the basis for the driving condition’s identification. Similar to speed prediction, regarding the uncertain route at an intersection, the slope prediction is modelled as a Markov model. On the basis of the predicted speed and the identified maximum adhesion coefficient, the slip ratio is predicted based on a neural network. Then, a predictive energy management strategy is developed based on the predictive full-factor trip information. According to the statistical rules of DP results under multiple standard driving cycles, the reference SOC trajectory is generated to ensure global sub-optimality, which determines the feasible state domain at each prediction horizon. Simulations are performed under different types of driving conditions (Urban Dynamometer Driving Schedule, UDDS and World Light Vehicle Test Cycle, WLTC) to verify the effectiveness of the proposed strategy.

## 1. Introduction

To cope with the problem of individual energy shortages and environmental pollution, developing new energy vehicles (NEVs) is an inevitable choice for the global automotive industry in the 21st century [1]. Plug-in hybrid elective vehicles (PHEVs) combine the advantages of traditional vehicles with pure electric vehicles (EVs), which is a conducive method to achieve energy security and carbon neutral goals. Structurally, the non-uniformity of energy sources makes hybrid vehicles more diverse in energy distribution when the power demand is the same. Thus, rationally, energy distribution is the key to researching the energy management of multi-energy source vehicles (MEVs).

At present, energy management control strategies for MEVs mainly divide into three categories: rule-based [2,3], optimization-based [4,5] and data-driven [6]. Reference [7] proposes a driving strategy based on fuzzy logic. Based on the logic threshold control, the predefined control rules are fuzzified by adding expert experience. The rule-based control strategy has been widely applied due to its simplicity and ease of implementation; however, it cannot obtain good fuel economy and realize the self-adaptation of driving conditions. To maximize fuel economy, global optimization control strategies have been developed, such as Pontryagin’s minimum principle (PMP) [8] and dynamic programming (DP) [9,10].

The dynamic programming strategy, as the representative global optimization method, can obtain the theoretical optimal fuel economy. Based on the dynamic programming algorithm, the trajectory of gears and engine on/off states are optimized in reference [11] to minimize the fuel consumption of vehicles driving on hilly roads. However, a DP-based strategy needs to acquire the whole driving information in advance. To overcome the difficulties of being put in to practice, it is essential to overcome the reliance on deterministic trip information and improve the computational efficiency.

The implementation of a DP strategy requires deterministic trip information in advance. Vehicle speed and slope, as the main trip information, has a significant impact on global optimization energy management. Under the Chinese Standard Urban Driving Cycle (CSUDC), reference [12] systematically studied the fuel-saving potential of the DP-based method. When we cannot obtain the speed information of the entire driving cycle in advance, the prediction of speed is necessary. There exist three methods to predict the vehicle speed, a Markov chain [13,14], artificial neural networks (NNs) [15,16] and exponentially varying prediction [17,18]. Whether it is a Markov chain velocity predictor or an NN-based velocity predictor, once there exists low similarity between real road conditions and training samples or history data, the prediction accuracy will significantly reduce.

Slope also has a significant impact on global optimization for energy management. Thanks to geographical information systems (GISs) and global positioning systems (GPSs) [19], the slope on a regular route is available. By considering the impact of road gradient on the driving demand of the vehicle, a plug-in hybrid electric vehicle energy management strategy based on road gradient information is proposed in reference [20]. However, the route to the destination is not unique for a flexible vehicle. During real driving, due to some unexpected situations, such as road congestion, traffic lights, etc., the driver will choose other routes. When the driver encounters multi-junction roads (such as crossroads or roundabouts), the driver’s motivation will affect the direction of the vehicle, that is, the slope cannot be directly obtained. In reference [21], the possible future routes, as well as their probabilities, are obtained using GPS information and historical driving data. Regarding the unknown future route, how to acquire the slope is the key to determining accurate power demand.

In addition, the trip information in the previous literature mainly involves the vehicle’s speed and slope, without considering the slip ratio. The slip ratio is related to wheel speed, which affects the calculation accuracy of power demand. In summary, comprehensive and accurate trip information should be acquired to provide an information foundation for global optimization energy management.

With the development of artificial intelligence (AI) and big data technology, data-driven strategy [22] has emerged on the basis of optimal controls, such as reinforcement learning (RL) [23,24] and adaptive dynamic programming (ADP) [25,26]. The core is to deeply mine DP behavior to achieve self-learning energy management; however, this is restricted by high device configuration and complex algorithms. Based on the predictive trip information, optimization in a short-term horizon can be achieved, which can significantly improve the real-time performance of the DP strategy. However, a reference state of charge (SOC) trajectory should be pre-planned to ensure global optimality. Thence, how to generate an approximate reference SOC trajectory is crucial for predictive energy management.

The target of this paper is to develop a predictive energy management strategy (PEMS) based on full-factor trip information, including vehicle speed, slope and slip ratio. First, the prediction model of the full-factor trip information is established. In particular, on the basis of the maximum adhesion coefficient identification model, the slip ratio is determined by the certain recurrent jump state network. Then, a predictive energy management strategy (PEMS) is developed, which generates a reference SOC trajectory to ensure global optimality. Finally, the influence of prediction time on prediction accuracy of the full-factor trip information is analyzed, which lays a foundation for determining the optimal prediction time to balance vehicle fuel economy and prediction accuracy.

The rest of this paper is organized as follows. The system architecture and powertrain modelling of PHEV is introduced in Section 2. The prediction model of the full-factor trip information is established in Section 3. Then, in Section 4, a predictive energy management strategy is developed. Two case studies (UDDS and WLTC) are performed to verify the effectiveness of the proposed strategy. Several conclusions are drawn in Section 5.

## 2. System Architecture and Modelling

### 2.1. PHEV Model

**Figure 1:**
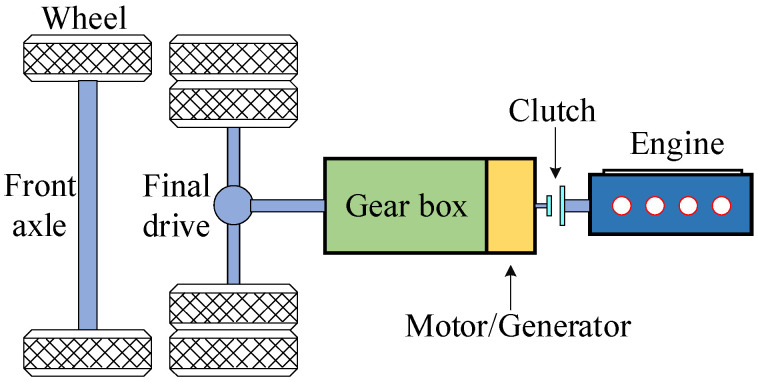
Schematic diagram of the PHEV drivetrain.

A plug-in hybrid electric vehicle (PHEV) with P2 configuration is taken as the research object, and its powertrain configuration is shown in Figure 1. The main component parameters of the PHEV are listed in Table 1.

According to the possible working state of each power component, this hybrid configuration can be roughly divided into three main modes: pure electric mode, series mode with disconnected clutch and parallel mode with closed clutch. Specifically, according to different patterns of energy flow, work modes can be further subdivided into the following modes, as shown in Table 2.

### 2.2. Vehicle Longitudinal Dynamics Model

In the longitudinal direction, the driving force in the driving process can be expressed as

(1) $$F_{D}=mgf\cos\alpha +\frac{C_{D}A\cdot v_{c}^{2}}{21.15}+mg\sin\alpha +\delta \cdot m\frac{du}{dt}$$

where $F_{D}$ is the driving force, m is the gross weight, g is the gravity acceleration, f is the rolling resistance coefficient, α is the road gradient, $C_{D}$ is the air resistance coefficient, A is the windward area, $v_{c}$ is the vehicle speed, δ is the rotation mass conversion factor and du/dt is the longitudinal acceleration of the vehicle. The main vehicle parameters of the PHEV model are summarized in Table 3.

### 2.3. Engine Model

The engine model is simplified as a quasi-static map to calculate the fuel consumption. That is, the fuel consumption is expressed as the relationship between the engine speed and engine torque

(2) $$Q_{e}=F_{fuel}\left(T_{e},n_{e}\right)$$

where $\;n_{e}$ is the engine speed, $T_{e}$ is the engine torque and $Q_{e}$ is the fuel consumption of the engine.

### 2.4. Electric Machine Model

The efficiency characteristics of motors can be formulated as

(3) $$\eta_{m}=f\left(T_{m},n_{m}\right)$$

where $\eta_{m}$ is the motor efficiency,$\;n_{m}$ is the motor speed and $T_{m}$ is the motor torque, which is defined as positive during propelling and negative during regenerative braking.

The output power $P_{m}$ of the drive motor can be written as

(4) $$P_{m}=\{\begin{array}{c} T_{m}\omega_{m}/\eta_{m},\;motor\\ T_{m}\omega_{m}\eta_{m},\;generator \end{array}$$

### 2.5. Power Battery Model

Without the consideration of temperature change and battery aging, an internal resistance battery model [27] is used to calculate the battery power. Ignoring thermal-temperature effects and battery transients, the state of charge (SOC) can be calculated by [28]

(5) $$dSOC=SOC_{t+1}-SOC_{t}=-\frac{U_{oc}-\sqrt{U_{oc}^{2}-4R_{int}\cdot P_{bat}}}{2R_{int}\cdot C}$$

where $SOC_{t+1}$ is the battery SOC at the moment t+1, and $SOC_{t}$, $U_{oc}$, $P_{bat}$ and $R_{int}$ are the SOC, open-circuit voltage, electric power and internal resistance of the battery at the moment t, respectively.

## 3. Prediction Model of Full-Factor Trip Information

Once the vehicle model is fixed, the vehicle’s own parameters are determined, and the longitudinal acceleration can be obtained by the derivative of velocity without considering lateral velocity.

According to the longitudinal dynamic equation, the calculation accuracy of power demand depends on the accuracy of the vehicle speed, slip rate and slope. The following will describe the prediction model for the above information.

### 3.1. Speed Prediction Based on the Improved Markov Model

#### 3.1.1. Generation of the State Transition Probability Matrix

The Markov process [29] proposed by Andrey Markov is a class of random processes. Within a certain period of time, the vehicle speed conforms to the Markov property. Regarding the speed and acceleration as coordinate axes, the state transition probability matrix of vehicle speed can be defined as

(6) $$T_{ij}=P\left[a_{k+m}={\bar{a}}_{j}|V_{k+m-1}={\bar{V}}_{i}\right]$$

where i∈{1,2,⋯,pp} is speed space, j∈{1,2,⋯,qq} is acceleration space, $V_{k+m-1}$ is the speed state at the moment k, $a_{k+m}$ is the acceleration state at the moment k+1, $m\in \left\{1,2,\cdots ,L_{p}\right\}$ and $L_{p}$ is the prediction time.

It should be emphasized that the above formula corresponds to the matrix span with 1 s. When the matrix span is n, the matrix is expressed as

(7) $$T_{ij}=P\left[a_{k+m}={\bar{a}}_{j}|V_{k+m-n}={\bar{V}}_{i}\right]$$

Due to the diversity of driving cycles, the speed prediction model with the unique state transition matrix will undoubtedly cause a large deviation. To improve the prediction accuracy, the types of driving cycles are distinguished, and multiple standard driving cycles under different types of driving cycles are regarded as the training samples to generate their respective state transition matrices.

**Figure 2:**
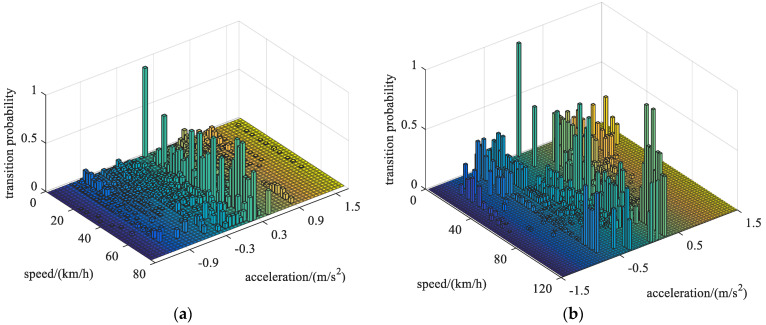

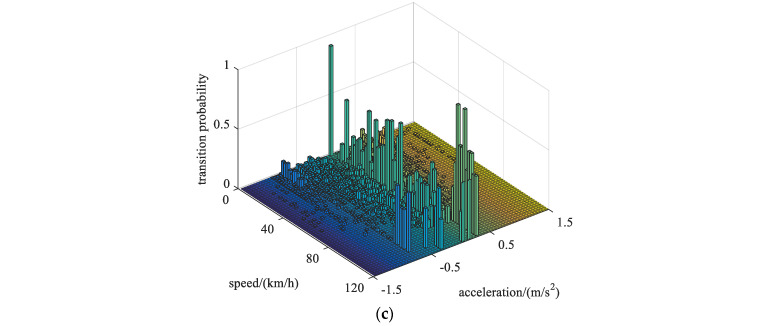
State transition probability matrix. (**a**) urban condition; (**b**) highway condition; (**c**) mixed condition.

By comparing some characteristic parameters of different driving cycles, they can be roughly divided into three categories: urban, highway and mixed conditions. Multiple standard driving cycles (including UDDS, UDC, EUDC, HWFET, NEDC, WLTC, etc.) are taken as the training samples to generate the state transition probability matrix by the maximum likelihood estimation method [30], corresponding to city, highway and mixed conditions, as shown in Figure 2.

#### 3.1.2. Selection of State Transition Matrix

Different from the traditional Markov model, there are three state transition matrices in the optimized model. Thence, one of the state transition probability matrices should be selected as the basis for speed prediction. Characteristic parameters are used to describe the characteristics of a substance or phenomenon. The characteristic parameters of different driving cycles have a significant difference, thence, the category can be judged according to the characteristic parameters of the driving conditions.

Physically, there exist 23 kinds of recognized characteristic parameters for vehicle driving cycles [31], as shown in Table 4.

Considering that most of the above characteristic parameters have little influence on classification, there may be redundant characteristic parameters, which will increase the calculation cost and is not conducive to rapid classification. Feature selection can eliminate irrelevant or redundant features to improve model accuracy and reduce runtime. Therefore, feature selection [32] is utilized to obtain a few characteristic parameters that have the greatest influence on classification results. The specific process of feature selection is as follows:

1. Generation Procedure

According to min–max standardization, all characteristic parameters are nondimensionalized in the range of (0,1). That is

(8) $${\hat{x}}_{i}=\frac{x_{i}-\min\left(X\right)}{\max\left(X\right)-\min\left(X\right)}$$

where X is the data set, $x_{i}\in X$ and ${\hat{x}}_{i}$ is the processed data.

2. Determine the Evaluation Function

Evaluation function is a criterion to evaluate the quality of a feature subset. Generally, the Laplacian score is used as the evaluation function to calculate the influence factors of each characteristic parameter on the driving cycle classification. The Laplace score [33] of the r−th feature is calculated as follows

(9) $$L_{r}=\frac{{\tilde{f}}_{r}^{T}\cdot L\cdot {\tilde{f}}_{r}}{{\tilde{f}}_{r}^{T}\cdot D\cdot {\tilde{f}}_{r}}$$

(10) $${\tilde{f}}_{r}=f_{r}-\frac{f_{r}^{T}\cdot D\cdot 1}{1^{T}\cdot D\cdot 1}$$

where $f_{r}={\left[f_{r1},f_{r2},\cdot \cdot \cdot ,f_{rm}\right]}^{T}$, $1={\left[1,\cdot \cdot \cdot ,1\right]}^{T}$,D=diag(S⋅1); $f_{ri}$ represents the i−th sample of the r−th feature, i=1,2,…,m, m is the number of features; diag(·) represents that a vector is transformed into a diagonal matrix.

The L is defined as

(11) L=D−S

(12) $$S_{ij}=\{\begin{array}{c} e^{-\frac{x_{i}-x_{j}{}^{2}}{t}},\;if\;d\left(x_{i},x_{j}\right)\leq k\\ 0\;\;\;\;\;\;\;\;\;\;\;\;\;\;\;\;\;,\;\;\;\;\;\;\;\;\;\;\mathrm{otherwise} \end{array}$$

where S is the weight matrix, t is an approximate constant and k is a distance constant to judge that the $x_{i}$ and $x_{j}$ are the same point. The distance d(x,y) is defined as

(13) $$d\left(x,y\right)=\sqrt{\left(\overset{m}{\underset{i}{\sum }}{\left(x_{i}-x_{j}\right)}^{2}\right)}$$

where i represents the characteristic sequence, $x\in \left(x_{1},x_{2},\ldots ,x_{m}\right)$, $y\in \left(y_{1},y_{2},\ldots ,y_{m}\right)$ and d(x,y) is the Euclidean distance between the point x and y.

3. Determine the Stopping Criterion

**Figure 3:**
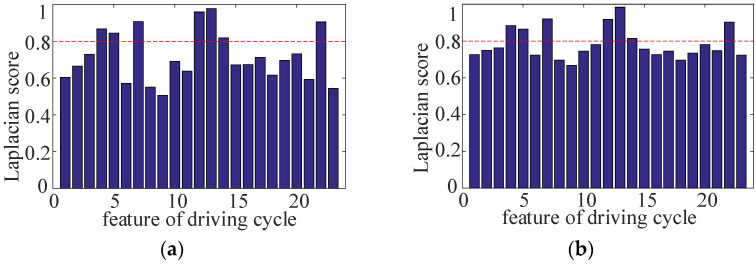
Laplacian scores and verification of characteristic parameters of driving cycles. (**a**) Laplace score of each feature; (**b**) verification.

When the value of evaluation function reaches the set threshold (stopping criterion), the search will be stopped. Taking multiple standard driving cycles as the samples, the Laplacian score of each feature is shown in Figure 3.

4. Validation Procedure

A new data set is utilized to verify the effectiveness of the selection.

Seven characteristic parameters with the higher Laplace score are selected as the judging basis of driving cycle recognition, which include average deceleration, average acceleration, the proportion of driving time at constant speed, deceleration time, standard deviation of acceleration/deceleration, driving time at constant speed and driving distance.

Then, a three-layer feed-forward neural network [34] with the structure of 7–7–1 is applied to classify driving conditions. The network inputs the characteristic parameters of the driving conditions and outputs the classification results (0/1/2), which correspond to urban, highway and mixed driving conditions. The initial weights of the network are set to be random in [−1, 1], and the number of neurons in the middle layer is obtained by the least squares method

(14) $$S=\sqrt{m\left(n+3\right)}+1$$

where S is the number of neurons in the middle layer, m is the number of neurons in the input layer and n is the number of neurons in the output layer.

**Figure 4:**
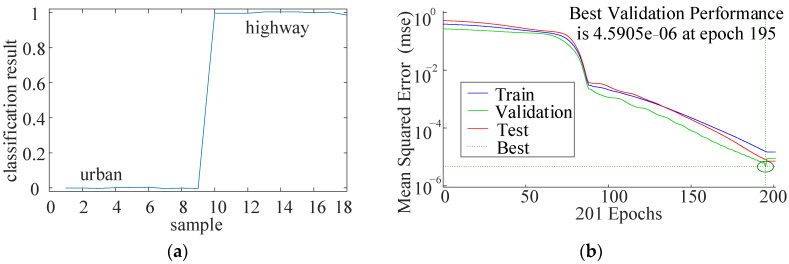
Classification results and verification. (**a**) Classification result; (**b**) Convergence of the BP network.

The training results and verification results are shown in Figure 4:

#### 3.1.3. Prediction of Speed Information

**Figure 5:**
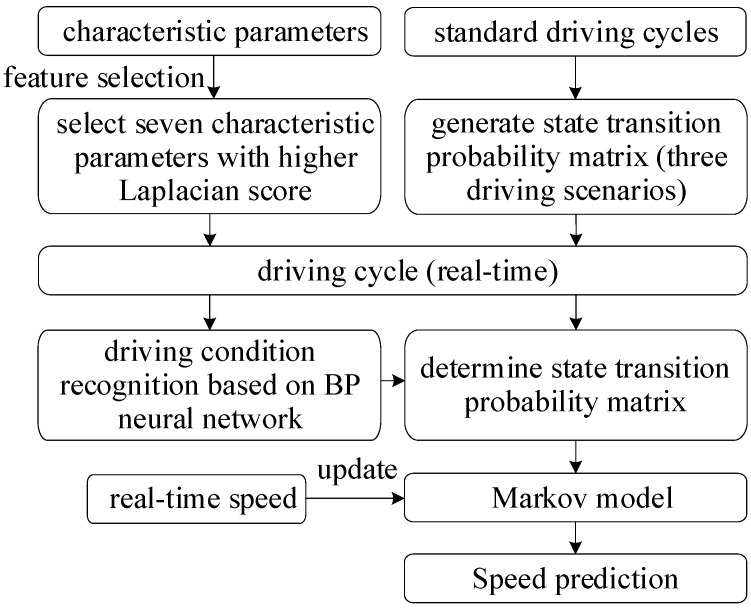
Overall process of speed prediction.

Once the type of driving cycle is identified, the corresponding state transition probability matrix will be utilized to realize the speed prediction. The overall process of speed prediction is shown as Figure 5.

**Figure 6:**
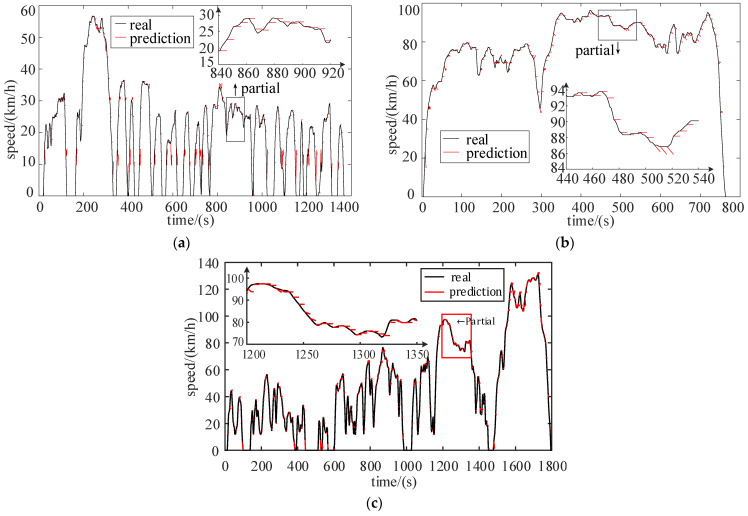
Speed prediction results. (**a**) Speed prediction of UDDS (urban); (**b**) Speed prediction of HWFET (highway); (**c**) Speed prediction of WLTC (mixed).

Taking UDDS, HWFET and WLTC cycles as examples, the speed at each moment is predicted based on the state transition probability matrix generated in the respective driving scenarios (urban, highway, mixed), and the prediction time is set to 5 s. The prediction results are shown in Figure 6.

Generally, the root-mean-square error (*RMSE*) is usually used to describe the prediction accuracy, which is expressed as

(15) $$RMSE=\sqrt{\frac{1}{N}\overset{N}{\underset{i=1}{\sum }}{\left(v_{i}^{\prime }-v_{i}\right)}^{2}}$$

where N is the step number of speed prediction, $v_{i}^{\prime }$ is the predictive speed and $v_{i}$ is the predictive speed.

Compared with the traditional Markov model (without driving cycle identification), the comparison of prediction accuracy is concluded in Table 5:

### 3.2. Slope Prediction Model

Considering that the route selection is consistent with the Markov property, the slope at multiple intersections can be predicted by the Markov model [35], which selects relative altitude and slope as state variables. Similar to velocity and acceleration, the relative altitude and slope are divided by intervals, which determine the corresponding relative altitude space as i∈{1,2,⋯,pp} and slope space as j∈{1,2,⋯,ss}. Thence, the state transition matrix of the road gradient is defined as

(16) $$T_{ij}=P\left[\theta_{k+1}={\overset{¯}{\theta }}_{j}|H_{k}={\bar{H}}_{i}\right]$$

where i∈{1,2,⋯,pp} is altitude space, j∈{1,2,⋯,ss} is slope space, $H_{k}$ is the relative altitude at the kth moment (or geographical location, the same below), $\theta_{k+1}$ is the slope at the (k+1)th moment.

The state transition matrix is constructed based on the relative altitude and slope within a certain range at intersections, which satisfies all constraints during driving. The length of the predicted range is determined by the speed and prediction time.

**Figure 7:**
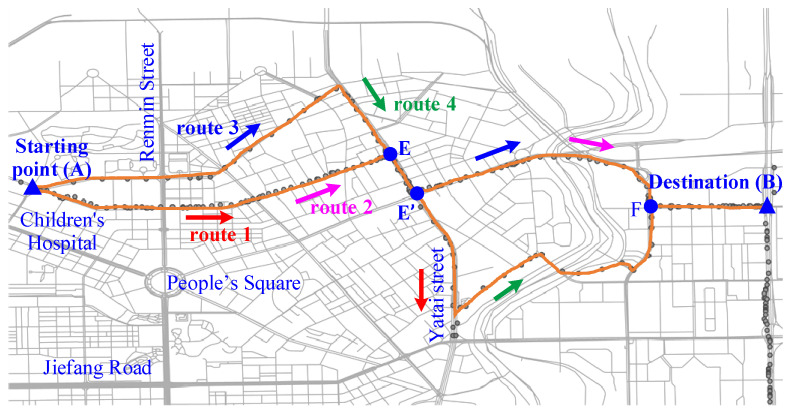
Schematic diagram of the area.

An area is taken as objective, as shown in Figure 7. The starting point is A, and the destination is B. The data are supported by NDANEV OPENLAB, which corresponds to the real-time driving data of EV-Bus 4 and PHEV-Bus 152 in Changchun city.

**Figure 8:**
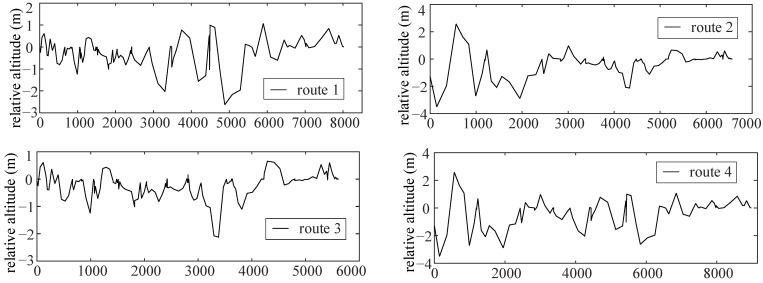
Relative altitude of the selected route.

All possible routes in this area and the corresponding absolute altitudes are obtained. By setting the reference point, the relative altitude and slope of each route in the area are acquired, which are shown in Figure 8.

**Figure 9:**
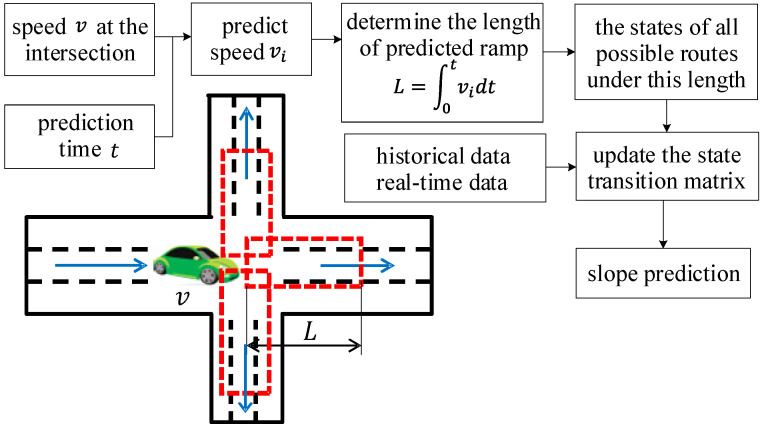
The flow diagram of slope prediction.

At each intersection, once the vehicle speed and prediction time are determined, the relative altitude and slope within the predicted range are regarded as samples to construct the state transition probability matrix for slope prediction. The specific process is shown in Figure 9.

**Figure 10:**
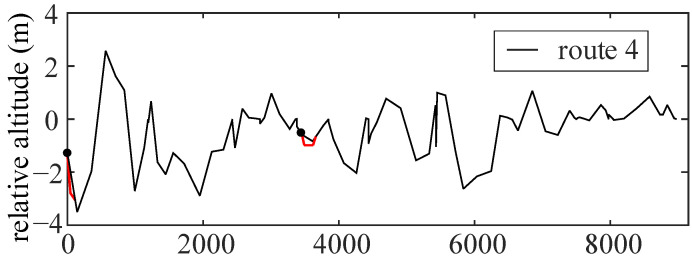
The simulation result of route 4.

Ensuring that the road conditions (features) are the same when simulating different driving cycles, the route 4 (green) is taken as an example to predict the slope, which includes two intersections (point A and E). The simulation results are shown in Figure 10.

Overall, the prediction deviation of the slope is not large.

### 3.3. Prediction Model of Slip Ratio

#### 3.3.1. Identification Model of the Maximum Adhesion Coefficient

The slip ratio is related to the road adhesion coefficient to some extent. The road adhesion coefficient μ can be formularized as a function of slip ratio and vehicle speed through the magic formula of the tire [36]. The specific formula is

(17) $$\mu \left(\lambda ,V\right)=\left(C_{1}\left(1-e^{C_{2}\lambda }\right)-C_{3}\lambda \right)e^{-C_{4}\lambda V}$$

where $C_{1},C_{2}$ and $C_{3\;}$ are the tire characteristic parameters on friction coefficient, which can be determined empirically when the vehicle is determined. $C_{4}$ is the influence parameter of vehicle speed on friction coefficient, which is usually 0.02–0.04.

**Figure 11:**
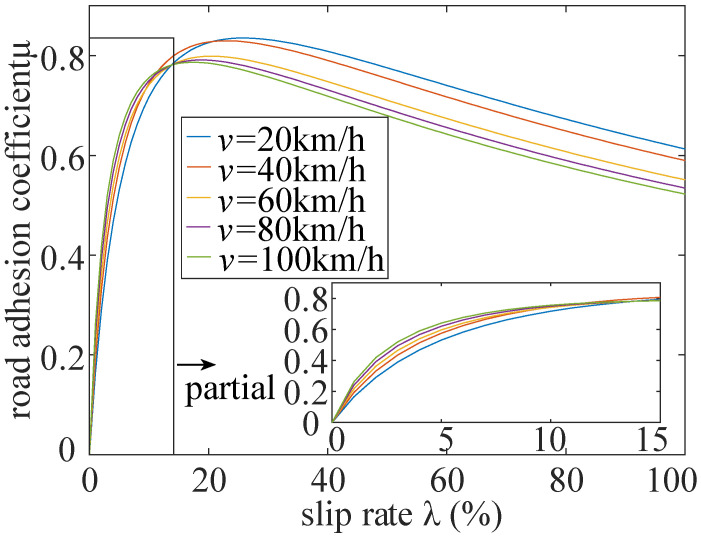
v-λ curve.

The effect of vehicle speed on the slip ratio is shown in Figure 11. 

**Figure 12:**
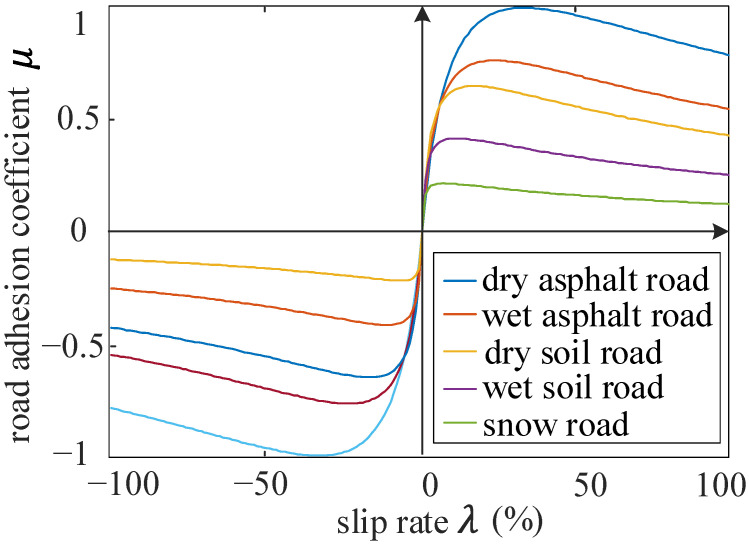
μ-λ of different road surface.

Based on previous research, the pavement material and road surface humidity are selected as influencing parameters to construct the identification model of the maximum adhesion coefficient. That is, the pavement roughness does not change when the pavement material is determined. When the vehicle speed is constant, the relationships between the adhesion coefficient and slip ratio of different road surfaces are shown in Figure 12. 

These can be summarized in the following conclusions:

a. When the vehicle speed is constant and the sliding ratio is small, the adhesion coefficient of each road surface is similar, which is difficult to distinguish;

b. When the sliding rate is about 20%, each road surface is close to the maximum adhesion coefficient;

c. With the increasing sliding rate, the road surface adhesion coefficient gradually decreases with a small reduction, and the adhesion coefficient is quite different, which is easy to distinguish.

Therefore, the slip ratio is segmented to construct the recognition model. The fuzzy recognition is adopted for the small sliding rate, while the large sliding rate needs to be accurately identified. Then, the slip ratio at a certain moment can be estimated with the predicted speed and road adhesion coefficient.

**Figure 13:**
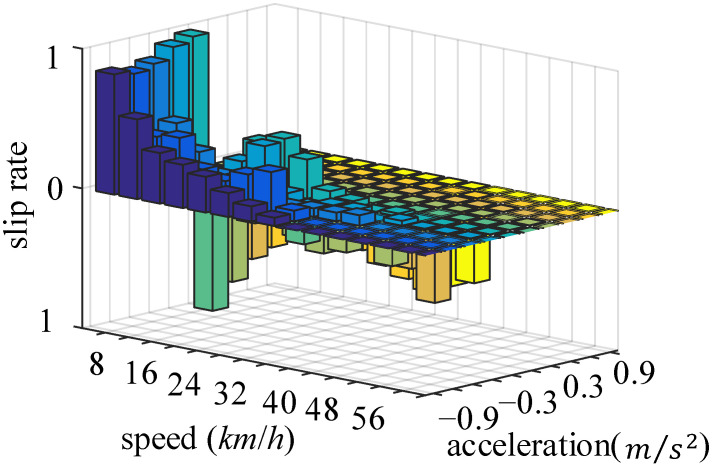
Database: wet asphalt matrix diagram under UDDS cycle.

Based on previous research, the pavement material and road surface humidity are selected as influencing parameters for road identification. Once the road type is determined, the vehicle speed has a large impact on the slip ratio, while having little effect on the maximum adhesion coefficient of the road surface. However, the slip ratio will change when the vehicle accelerates or decelerates. Therefore, the vehicle’s speed and acceleration/deceleration are taken into account to build an offline database. Taking wet asphalt pavement as an example, the matrix diagram is shown in Figure 13.

When the slip ratio is small, the adhesion coefficients of various pavements are relatively close, and are difficult to distinguish. To improve the prediction accuracy, the slip ratio is divided into two categories: the interval of small slip ratio (the slip ratio below 10%) and the interval of large slip ratio (the slip ratio above 10%).

**Figure 14:**
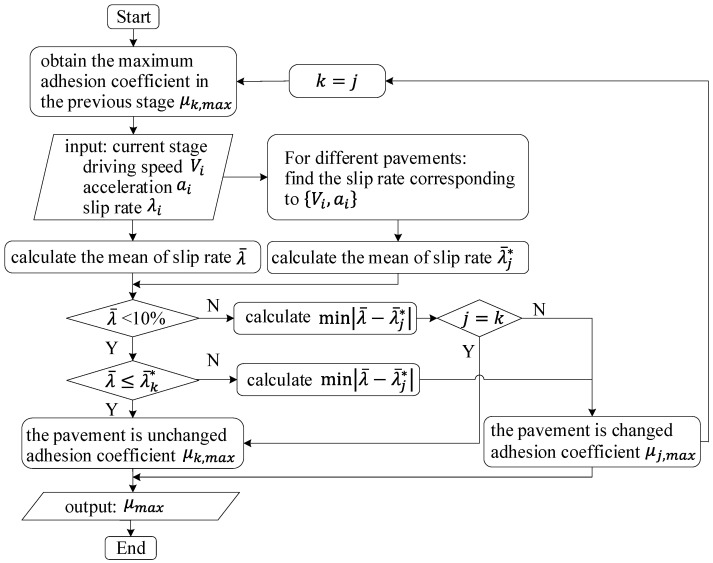
Identification process of $\mu_{\max}$ of pavement.

With respect to the large slip ratio, the adhesion coefficient of each pavement can be clearly distinguished. Comparing the input (slip ratio) with each interval, the road type can be determined by the minimum deviation method to obtain the maximum adhesion coefficient. For the small slip ratio, the deviation between the input and the slip ratio of each pavement type can be calculated. If the deviation at the previous moment is less than the mean of all deviations, the road type is considered unchanged. On the contrary, if it is greater than the mean value, the road type corresponding to the smallest deviation is taken as the identified pavement type, and the maximum adhesion coefficient is determined accordingly. The specific process is shown in Figure 14.

**Figure 15:**
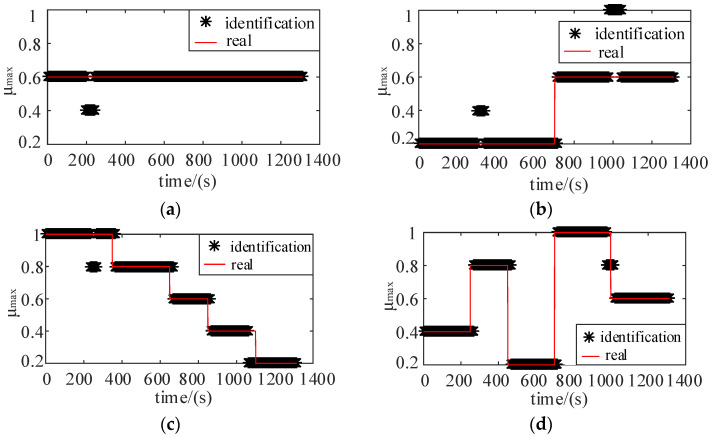
(**a**) Single pavement; (**b**) Combination of double pavement; (**c**) Multi-road combination, stepped pavement; (**d**) Multi-road combination, fully mixed pavement (including 1.0/0.8/0.6/0.4/0.2).

By setting the corresponding parameters, different combinations of pavements are identified, as shown in Figure 15:

The specific recognition accuracy of each pavement is shown in Table 6. It can be seen that the model can better identify the maximum adhesion coefficient of the road surface, which can provide the main parameter for the construction of the slip ratio prediction model.

#### 3.3.2. The Prediction Model of Slip Ratio Based on Back Propagation (BP) Neural Network

Considering the convenience of parameter adjustment, a simple back propagation neural network (BPNN) is utilized to achieve the prediction of the slip ratio. A three-layer feed-forward neural network with the structure of 3–5–1 is established to predict the slip ratio. The vehicle speed, maximum adhesion coefficient of the pavement and slip ratio in the previous stage are taken as inputs of the BP network, and it outputs the predicted slip rate at the current stage.

**Figure 16:**
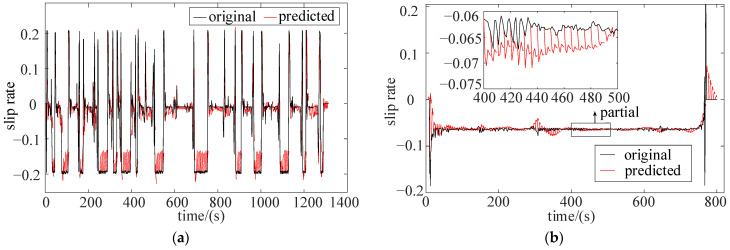
Slip ratio prediction results. (**a**) UDDS; (**b**) HWFET. (single pavement).

Under different types of driving cycles, multiple standard driving cycles are used to train the BP network. The prediction horizon is set to 5 s, and the simulations are performed under UDDS and HWFET cycles, as shown in Figure 16.

Similar to the speed prediction, the RMSE is used to describe the prediction accuracy. When the prediction time is 5 s, the RMSE under UDDS and HWFET is 0.091 and 0.07, respectively. Due to the fact that the BPNN can achieve a good prediction accuracy, the BP network is utilized to predict the slip rate.

## 4. Predictive Energy Management Based on Predictive Trip Information

### 4.1. Predictive Energy Management Strategy Based on Predictive Trip Information

With a fixed vehicle configuration, simulations with different SOC ranges are performed. By analyzing the change trend of the optimal SOC trajectory, we found that the optimal SOC trajectory obtained by the DP strategy declines linearly as a whole.

Thus, the terminal SOC at each prediction horizon can be preliminarily determined by the linear decreasing rule. That is, a reference SOC ($SOC_{r}$) is defined as

(18) $$SOC_{r}\left(j\right)=SOC_{0}-\frac{SOC_{0}-SOC_{f}}{N}\cdot j$$

where $SOC_{0}$ is the initial SOC, $SOC_{f}$ is the terminal SOC, N is the total driving time and j is the moment.

In particular, when the vehicle is parking, the reference SOC trajectory will be re-planned according to the linear decreasing rule. Moreover, when the vehicle can only recover energy, the terminal SOC of each prediction horizon will be re-planned according to the regenerative braking mode (pure electric mode). The energy recovery rate is set to 30%.

**Figure 17:**
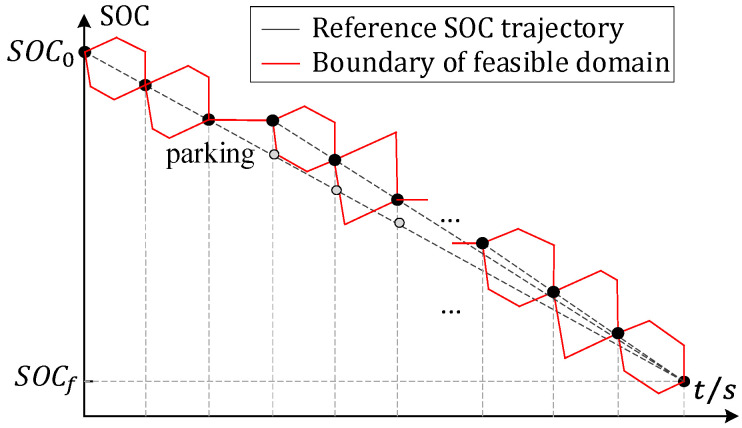
Schematic diagram of state feasible domain at each prediction horizon.

For the different speed distributions, the fluctuation degree of the optimal SOC trajectory relative to the reference SOC trajectory is different. Thence, the schematic diagram of the state feasible domain is shown in Figure 17.

At each prediction horizon, based on the predicted trip information, the optimal control can be determined based on the DP method. In the DP space model, the state equation of the PHEV model can be generally expressed as

(19) $$\{\begin{array}{c} x_{k+1}=f\left(x_{k},u_{k}\right)\\ x=\left[v\;SOC\right]\\ u=\left[T_{e}\;T_{m}\right] \end{array}$$

where x denotes the state variables, u denotes the control variables, k=0,1,2,…,l−1, and l is the prediction time.

The objectives of the deterministic dynamic programming (DDP) in PHEVs are to obtain the optimal SOC trajectory and minimize fuel consumption over a given driving cycle. That is, the optimal cost function of each prediction horizon can be regarded as the minimum fuel consumption for that stage. Consequently, the cost function can be expressed as

(20) $$J=\overset{l-1}{\underset{k=0}{\sum }}U\left(x_{k},u_{k}\right)=\overset{l-1}{\underset{k=0}{\sum }}fuel_{k}$$

where U denotes the instantaneous cost at each moment and $fuel_{k}$ denotes the instantaneous fuel consumption of each stage.

To ensure safe operation of the components (such as the engine, motors and power battery), the physical constraints on state variables and control variables are necessary during the optimization, that is

(21) $$\{\begin{array}{c} SOC_{\min}\leq SOC\left(k\right)\leq SOC_{\max}\\ T_{e\_\min}\leq T_{e}\left(k\right)\leq T_{e\_\max}\\ n_{e\_\min}\leq n_{e}\left(k\right)\leq n_{e\_\max}\\ T_{m\_\min}\leq T_{m}\left(k\right)\leq T_{m\_\max}\\ n_{m\_\min}\leq n_{m}\left(k\right)\leq n_{m\_\max} \end{array}$$

where the subscripts min and max denote the maximum and minimum of the corresponding variables, respectively.

### 4.2. Application

#### 4.2.1. The Influence of Prediction Time on the Prediction Accuracy

Based on the proposed prediction model, the prediction accuracy of each trips’ information at different prediction times is concluded in Table 7.

Regarding the speed prediction model, regardless of the driving conditions, as the prediction time increases, the prediction accuracy decreases significantly. For the slip ratio prediction model, when the prediction time is small, the prediction accuracy decreases significantly as the time scale increases; however, when the prediction time is large, the prediction accuracy is less affected by the prediction horizon (prediction time). With respect to the slope prediction model, the prediction accuracy for urban conditions is better than for highway conditions. The reason for this phenomenon is mainly due to the high vehicle speed under highway conditions, which increases the length required for slope prediction and increases the accumulated errors. With the increase in the prediction time, the prediction accuracy of the slope under the different driving conditions gradually decreases.

Under different prediction times, the predictive energy management strategy is developed based on the predictive full-factor trip information. Taking 10 s prediction time as an example, the simulations are implemented under different types of driving operation conditions (UDDS and WLTC) based on MATLAB software.

#### 4.2.2. Simulation Analysis of Urban Condition (UDDS)

**Figure 18:**
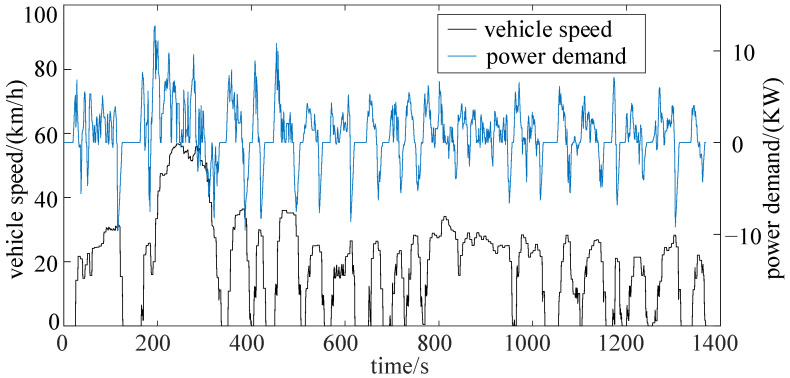
Vehicle speed and power demand of UDDS cycle.

The UDDS cycle is a typical urban driving cycle. The prediction time is set to be 10 s, the predicted speed and the power demand are shown as Figure 18. The maximum speed does not exceed 60km/h, and the power demand is between −10KW and 10KW.

**Figure 19:**
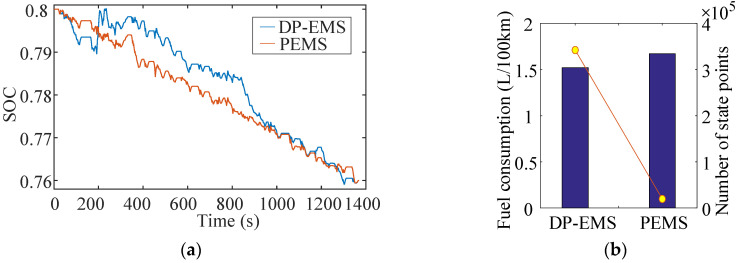

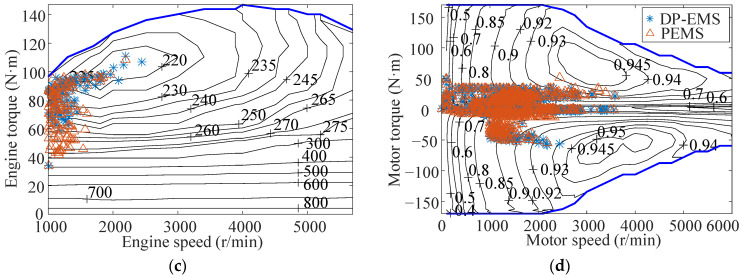
The simulation results of 10 s time scale under UDDS cycle. (**a**) Battery SOC; (**b**) Fuel consumption; (**c**) Engine; (**d**) Motor.

Setting the boundary of SOC to 76–80%, the simulation results of the 10 s prediction horizon under the UDDS cycle are shown in Figure 19.

Due to the low speed and lower power demand, the engine mainly works in low engine speed (1000~2000 r/min), and low engine torque (40~90 N·m). Meanwhile, the motor mainly operates in low motor torque (−50~50 N·m) and low motor speed (0~3000 r/min). The fuel consumption under a UDDS cycle is 0.091kg, and the corresponding fuel consumption per 100 km is 1.6626 L/100km. Compared with DP strategy, the fuel economy of the vehicle is reduced by 9.5%, while the real-time performance (characterized by the number of state points) is increased by more than 15 times.

#### 4.2.3. Simulation Analysis of Mixed Condition (WLTC)

**Figure 20:**
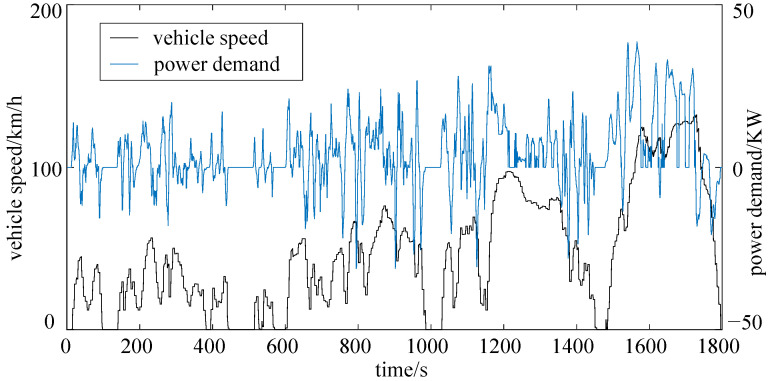
Vehicle speed and power demand of WLTC cycle.

The WLTC cycle is regarded as a combined cycle of urban and highway driving, as shown in Figure 20.

**Figure 21:**
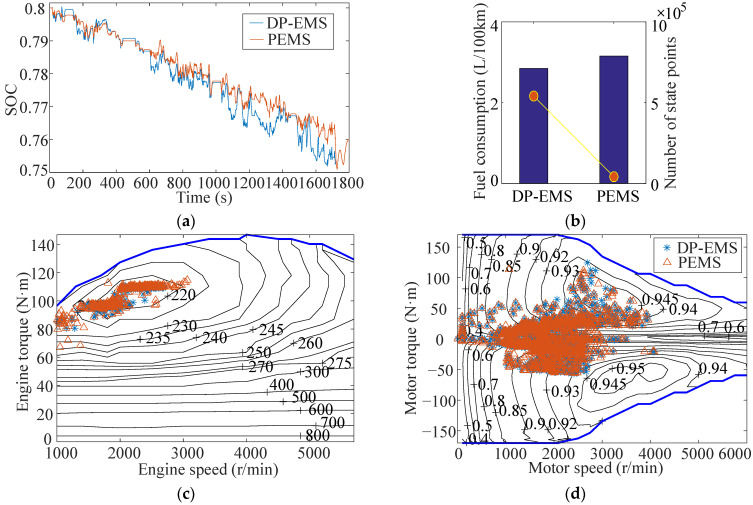
The simulation result of 10 s time scale under WLTC cycle. (**a**) Battery SOC; (**b**) Fuel consumption; (**c**) Engine; (**d**) Motor.

The maximum speed does not exceed 140km/h, and the power demand is between −40 KW and 40 KW. Under the predictive energy management control strategy and DP strategy, the simulation results following the WLTC cycle are shown in Figure 21.

The engine mainly operates in the middle engine speed (80~110 N·m) and low engine speed (1000~3000 r/min), while the motor mainly operates in the middle motor speed (1000~4000 r/min) and middle motor torque (−70~90 N·m). The fuel consumption under a WLTC cycle is 0.53945kg, and the corresponding fuel consumption per 100 km is 3.1551L/100 km. Compared with the DP strategy, the fuel economy of the vehicle is reduced by 10%, while the real-time performance (characterized by the number of state points) is increased by more than 10 times.

The simulations under different types of driving condition show that the proposed strategy improves the vehicle economy while improving the global optimality as much as possible. In the following research, determining the optimal prediction time to balance a vehicle’s fuel economy and prediction accuracy is our research focus, which can provide a theoretical basis for determining the optimal frequency of information collection for intelligent connected vehicles.

## 5. Conclusions

Dynamic programming (DP), as a typical global optimization method, can obtain the theoretical optimal fuel economy of multi-energy source vehicles (MEVs). To overcome the difficulties in the practical application of a traditional DP control strategy, a predictive energy management strategy (PEMS) is proposed based on the predictive trip information.

With the Intelligent Connected Vehicle (ICV) and Intelligent Transportation System (ITS), the data from the “drives–vehicles–roads” system are available. Based on the basic information, the prediction model of full-factor trip information is established. To be specific, combined with driving condition recognition, the vehicle speed is predicted based on the state transition probability matrix. Similarly, the slope at intersections is predicted based on the Markov model. On the basis of the above, the slip ratio is determined based on the identified maximum adhesion coefficient. Then, a predictive energy management strategy is developed, which pre-plans the terminal SOC of each prediction horizon to ensure the optimality. The reference SOC is determined by exploring the trend of the optimal SOC trajectories under multiple standard driving cycles.

To verify the effectiveness of the proposed model, two case studies (UDDS and WLTC) are given. The simulation results verify that the proposed strategy improves the vehicle economy while improving the global optimality as much as possible.

## Figures and Tables

**Table 1:** Basic parameters of the PHEV powertrain.

Component	Description	Value
Engine	displacement	1.6l
	maximum power	77.2 kW @ 5700 r/min
	maximum torque	147 N·m @ 4000 r/min
Motor	maximum power/torque	44.5 kW/170 N·m
Battery pack	nominal voltage/capacity	360 V/8.9 kWh
Gear box	gear	(3.867;2.217;1.371;0.930;0.956;0.767)

**Table 2:** The main work modes of PHEV with P2 configuration.

Number	Engine	Motor	Clutch	Work Mode
1	×	electric	off	pure electric mode
2	×	generate	off	regenerative braking mode
3	√	×	on	engine-only mode
4	√	electric	on	hybrid mode
5	√	generate	on	recharging mode
6	×	×	off	parking mode/sliding mode

**Table 3:** Main vehicle parameters of the PHEV model.

Vehicle Parameters	Value
Curb mass (kg)	1500
Wheel radius (mm)	306.94
Windward area ($m^{2}$)	2.25
Rolling resistance coefficient	0.012
Aerodynamics	0.24

**Table 4:** Main vehicle parameters of the PHEV model.

Num	Features	Unit	Num	Features	Unit
1	driving time	s	2	maximum speed	km/h
3	acceleration time	s	4	deceleration time	s
5	uniform speed time	s	6	idle time	s
7	driving distance	m	8	average speed	km/h
9	standard deviation of speed	km/h	10	maximum acceleration	m/s^2^
11	maximum deceleration	m/s^2^	12	average acceleration	m/s^2^
13	average deceleration	m/s^2^	14	standard deviation of acceleration/deceleration	m/s^2^
15	speed proportion (0–10 km/h)	/	16	speed proportion (10–20 km/h)	/
17	speed proportion (20–30 km/h)	/	18	speed proportion (30–40 km/h)	/
19	speed proportion (40–50 km/h)	/	20	time proportion (acceleration)	/
21	time proportion (deceleration)		22	time proportion (uniform speed)	/
23	time proportion (idle)	/			

**Table 5:** The comparison of predictive accuracy (prediction time = 5 s).

Cycles	Time	Optimization	*RMSE*	Optimization Rate (%)
UDDS	1369	before	4.8065	7.1
		after	4.4637	
HWFET	766	before	3.4524	6.5
		after	3.2282	
WLTC	1800	before	5.7912	6.2
		after	5.4326	

**Table 6:** Recognition accuracy of different pavements.

Pavement	Pavement Type	Change Frequency	Recognition Accuracy (%)
single pavement	1	before	96
double pavement	2	after	95
stepped pavement	5	before	89
fully mixed pavement	4	after	82

**Table 7:** Prediction accuracy of each trips’ information at different prediction times under UDDS and WLTC cycles.

*RMSE*	Cycle	Prediction Time				
		1 s	5 s	10 s	20 s	30 s
vehicle speed *	UDDS	1.7315	4.4637	7.0765	11.2392	13.3799
	WLTP	1.9084	5.4326	8.9939	12.9163	15.3635
slope	UDDS	/	0.3423	0.4141	0.4214	0.4227
	WLTP	/	1.1497	1.2339	1.3007	1.3560
slip ratio *	UDDS	0.0511	0.0887	0.0947	0.1066	0.1077
	WLTP	0.0396	0.0517	0.0602	0.0709	0.0751

* The pavement type is single pavement; the span of state transition probability matrix is 1 s.
